# Supplementary material for: The Interplay between Colon Cancer Cells and Tumour-Associated Stromal Cells Impacts the Biological Clock and Enhances Malignant Phenotypes
Source: Cancers (Basel). 2019 Jul 15;11(7):988. doi: 10.3390/cancers11070988 (PMC6678177; doi:10.3390/cancers11070988)
Supplement: Supplementary file 1 [file cancers-11-00988-s001.pdf]

Supplementary Materials: The Interplay between Colon Cancer Cells and Tumour-Associated Stromal Cells Impacts the Biological Clock and Enhances Malignant Phenotypes

Luise Fuhr, Monica Abreu, Annalucia Carbone, Rukeia El-Athman, Fabrizio Bianchi, Mikko O. Laukkanen, Gianluigi Mazzoccoli and Angela Relógio

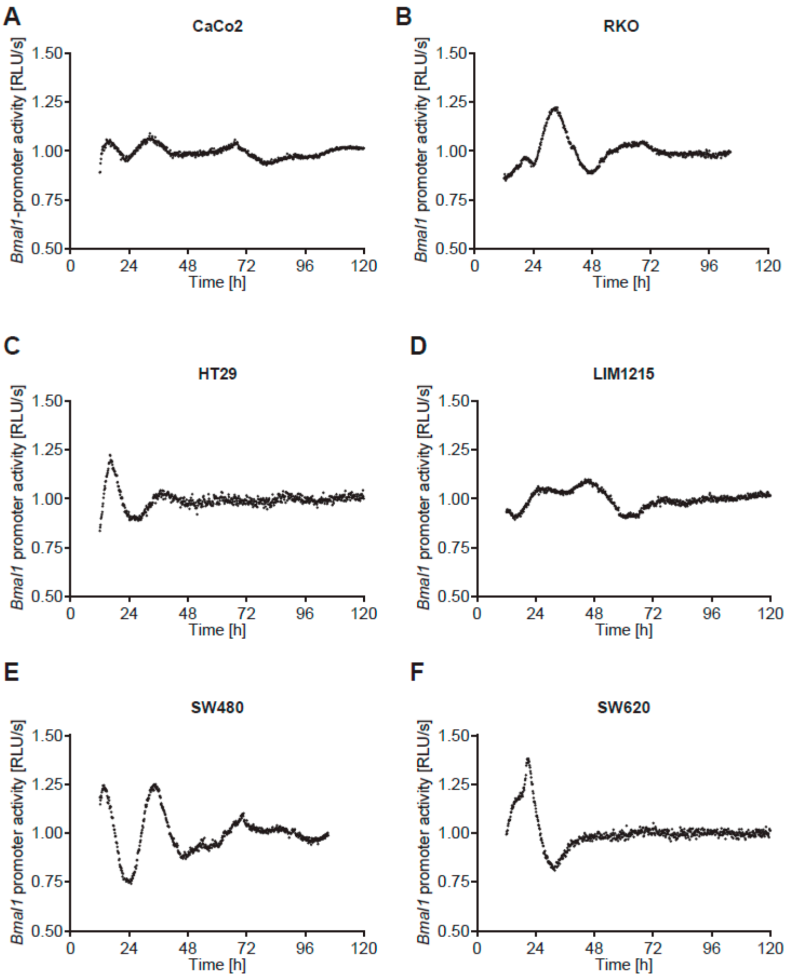

Figure S1. Clock phenotypes of different colorectal cancer cell lines. Bioluminescence measurements of Caco2 (**A**), RKO (**B**), HT29 (**C**), LIM1215 (**D**), SW480 (**E**) and SW620 (**F**) cells. Cells were lentivirally transduced with a Bmal1-luciferase construct (BLH) and bioluminescence was measured over five days. Displayed is one representative replicate per cell line.

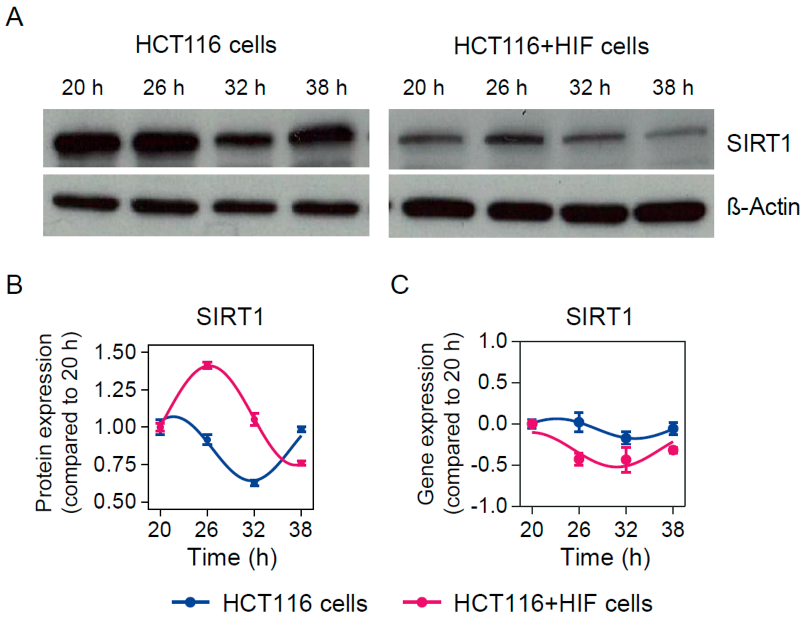

Figure S2. Co-culture of HCT116 and HIF cell lines alters the rhythmic expression of SIRT1. (**A**) Time-series Western Blotting of the proteins SIRT1 and β-Actin. (**B**) Time-series expression profiles of the protein SIRT1 in HCT116 cells (dark blue, p = 9.94e-10, acrophases = 1.7 h, amplitude = 0.24) and a HCT116+HIF co-culture (pink, p = 6.85e-06, acrophases = 4.3 h, amplitude = 0.31). A sine-cosine curve was fitted to the data using the model $y = m + a * \sin\left(2 * \pi * \frac{t}{\omega}\right) + b * \cos\left(2 * \pi * \frac{t}{\omega}\right)$. Period ω for the different conditions was chosen dependent on the period lengths observed in the long term real-time bioluminescence recording of *BMAL1* promoter activity (HCT116: 20 h, HCT116+HIF: 22 h). (C) Time-series expression profiles of the gene *SIRT1* in HCT116 cells (dark blue) and a HCT116+HIF co-culture (pink) (see also Figure 2).

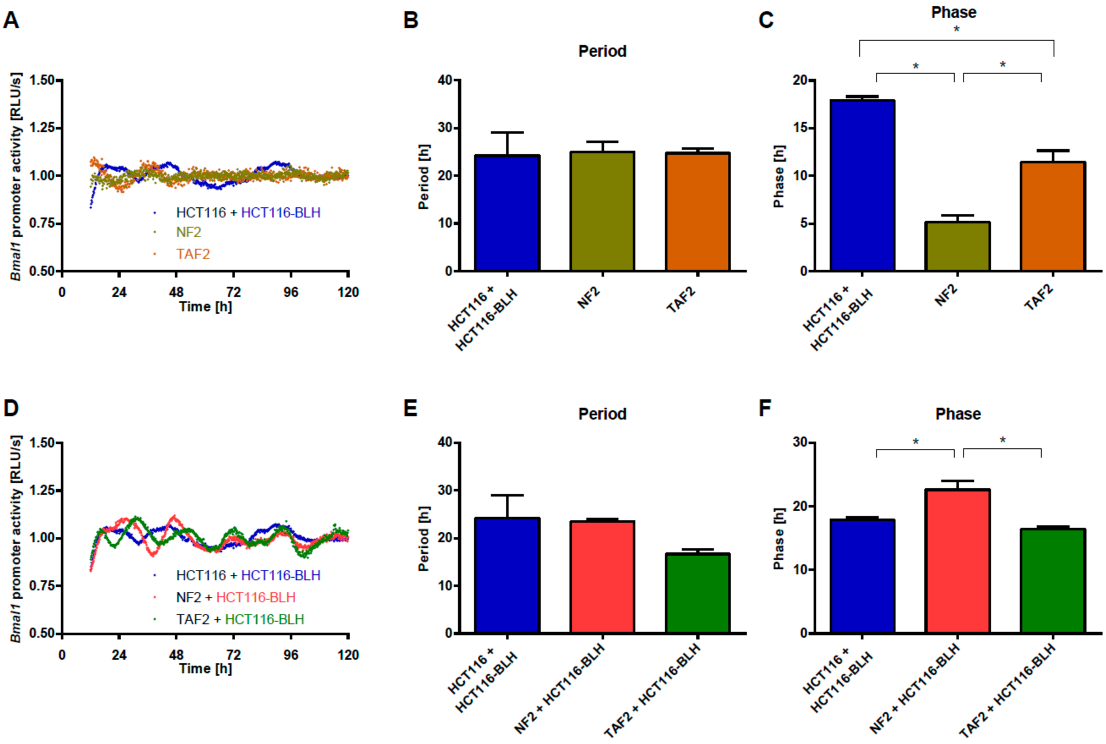

Figure S3. Cell-to-cell communication impacts on the circadian phenotype. (**A**) HCT116, NF2 and TAF2 cells were lentivirally transduced and the BMAL1-promoter activity was measured over five consecutive days. Shown is one representative replicate per condition. Period (**B**) und phase (**C**) were calculated. Data are expressed as mean ± SEM, n = 3. Significant changes ($p < 0.05$) between different

cells are marked with *. (**D**) HCT116, NF2 and TAF2 cells were lentivirally transduced and the BMAL1-promoter activity was measured over five consecutive days. HCT116 cells were either co-cultured with themselves or with NF2s or TAF2s. Shown is one representative replicate per condition. The sample written in coloured letters was the one that was measured. Period (**E**) und phase (**F**) were calculated. Data are expressed as mean ± SEM, $n = 3$. Significant changes ($p < 0.05$) between different conditions are marked with *.

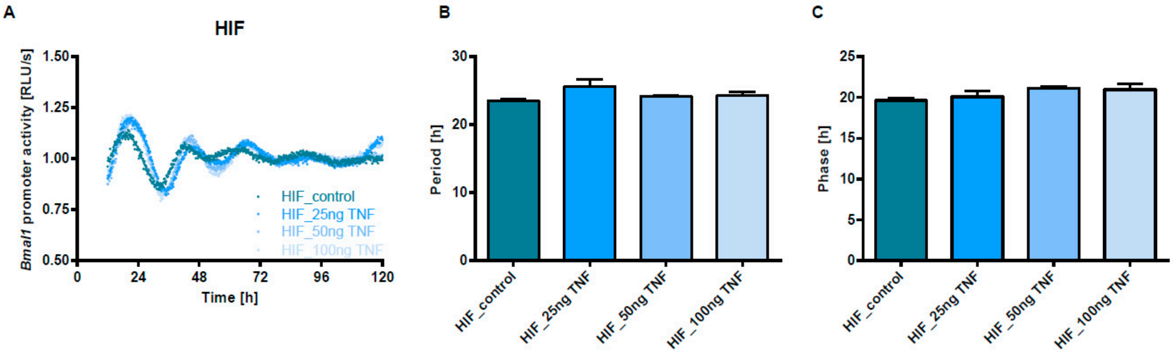

Figure S4. Effect of the stimulation with increasing amounts of human recombinant TNF on circadian rhythms in HIF cell lines. HIF lentivirally transduced cells were cultured with increasing concentrations of human recombinant TNF (25 ng/mL, 50 ng/mL and 100 ng/mL), and the *BMAL1*-promoter activity was measured over five consecutive days. (**A**) Shown is one representative replicate per condition. Colour gradients represent the different concentrations of recombinant human TNF used. Period (**B**) and phase (**C**) were calculated in the samples and comparisons were made to the control condition. Data are expressed as mean ± SEM, $n = 3$. Significant changes ($p < 0.05$) between different conditions are marked with *.

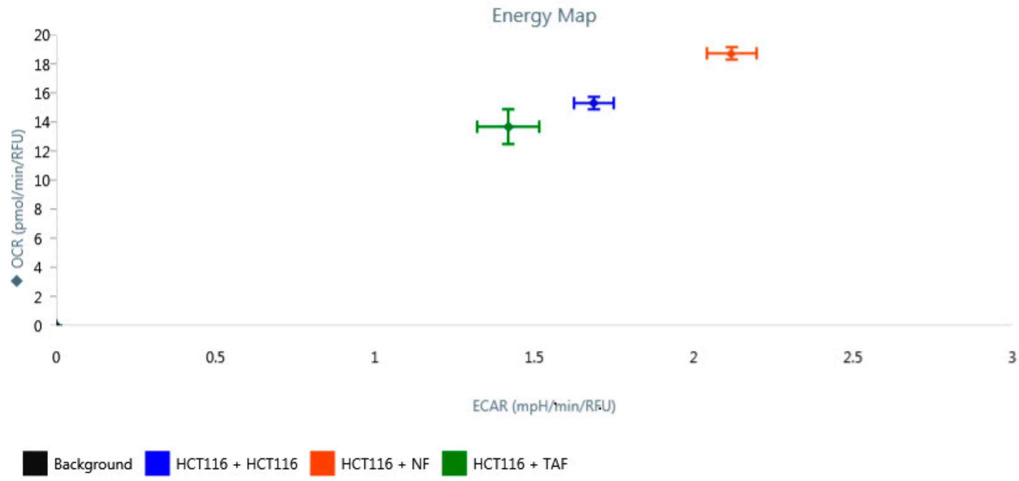

Figure S5. Energy map for HCT116 cells showing the co-culture effect caused by NFs or TAFs. Mean ± SEM, $n = 6$.

Table S1. Harmonic regression analysis of rhythmicity for core-clock genes.

Gene	Condition	Period [hours]	*p*-Value	Acrophase [hours]	Amplitude	Acrophase Difference (HCT116_HIF-HCT116) [hours]
BMAL1	HCT116_HIF	22	0.1512	13.6	0.29	−1.6
BMAL1	HCT116	20	0.2204	15.2	0.38	
BMAL1	HIF	25	0.2515	21.6	0.30	
BMAL2	HCT116_HIF	22	0.1633	14.1	0.61	9.5
BMAL2	HCT116	20	0.4072	4.5	0.19	
BMAL2	HIF	25	0.6698	18.8	0.33	
CLOCK	HCT116_HIF	22	0.4059	16.2	0.56	7.0
CLOCK	HCT116	20	0.5541	9.2	0.19	
CLOCK	HIF	25	0.9763	14.9	0.01	
CMYC	HCT116_HIF	22	0.1202	19.0	0.20	15.5
CMYC	HCT116	20	0.0264	3.4	0.30	
CMYC	HIF	25	0.3462	20.0	0.60	
CRY1	HCT116_HIF	22	0.2363	13.7	0.36	5.9
CRY1	HCT116	20	0.0048	7.8	0.32	
CRY1	HIF	25	0.8096	17.8	0.20	
CRY2	HCT116_HIF	22	0.2028	15.2	0.47	10.4
CRY2	HCT116	20	0.7582	4.8	0.14	
CRY2	HIF	25	0.6761	17.4	0.26	
CSNK1E	HCT116_HIF	22	0.2312	13.7	0.64	12.6
CSNK1E	HCT116	20	0.7986	1.1	0.14	
CSNK1E	HIF	25	0.7051	18.2	0.40	
NR1D1	HCT116_HIF	22	0.2151	18.1	0.40	0.2
NR1D1	HCT116	20	0.2745	17.9	0.50	
NR1D1	HIF	25	0.7818	17.4	0.16	
PER1	HCT116_HIF	22	0.3946	14.9	0.56	-0.5
PER1	HCT116	20	0.6125	15.5	0.37	
PER1	HIF	25	0.3462	18.8	0.54	
PER2	HCT116_HIF	22	0.1255	17.1	0.41	9.8
PER2	HCT116	20	0.2761	7.4	0.35	
PER2	HIF	25	0.609	18.8	0.26	
PER3	HCT116_HIF	22	0.0006	14.6	0.85	11.1
PER3	HCT116	20	0.6378	3.5	0.73	
PER3	HIF	25	0.4581	19.2	0.59	
RORA	HCT116_HIF	22	0.1678	19.5	0.34	14.9
RORA	HCT116	20	0.7563	4.6	1.72	
RORA	HIF	25	0.4585	19.9	3.52	
SIRT1	HCT116_HIF	22	0.5095	19.8	0.21	16.7
SIRT1	HCT116	20	0.0728	3.2	0.12	
SIRT1	HIF	25	0.1054	21.7	0.40	
TIMELESS	HCT116_HIF	22	0.2884	20.2	0.11	
TIMELESS	HCT116	20	0.7757	17.1	0.04	
TIMELESS	HIF	25	0.4275	21.1	0.44	
TIPIN	HCT116_HIF	22	0.427	20.2	0.36	14.6
TIPIN	HCT116	20	0.4176	5.6	0.23	
TIPIN	HIF	25	0.161	22.8	0.47	
WEE1	HCT116_HIF	22	0.6445	0.1	0.13	−15.7
WEE1	HCT116	20	0.3199	15.8	5.45	
WEE1	HIF	25	0.091	22.6	0.40	

© 2019 by the authors. Licensee MDPI, Basel, Switzerland. This article is an open access article distributed under the terms and conditions of the Creative Commons Attribution (CC BY) license (http://creativecommons.org/licenses/by/4.0/).
